# Sewer Gas and Its Effects upon Health

**Published:** 1882-09

**Authors:** Byron W. Griffin

**Affiliations:** Lecturer on Etiology and Hygiene, in the Woman’s Medical College, of Chicago; 257 West Madison St.


### Original Communications.

---

ARTICLE II.

SEWER-GAS, AND ITS EFFECTS UPON HEALTH. A Lecture by BYRON W. GRIFFIN, M.D., Lecturer on Etiology and Hygiene, in the Woman's Medical College, of Chicago.

No one thing so much affects our physical well being as the air we breathe. Probably not one person in ten thousand is surrounded by perfect atmospheric conditions. The air is rendered impure by many causes. But it is safe to say that, among the harmful influences which can be named, none occupies a more important place than sewer-gas. "It is the *bête noire* of town residences." Many of the diseases which attack and destroy life among dwellers in cities are the result, either directly or indirectly, of this noxious agent, commonly known as sewer-gas. Sanitarians have already reached that place where, if the code of health laws they have framed could be promptly and efficiently enforced, many of the diseases now enumerated in the nomenclature of the medical man, would entirely disappear. Think, for a moment, of the large number of etiological factors entering into the causation of the different diseases met with in a large city. Adulterated food, overcrowding, filthy streets and alleys, improperly constructed dwellings, vitiated air, all these combine to swell the grand total of sickness and wretchedness existing in most large cities. Very few people, comparatively, take these causes of disease into account. Very few persons appreciate the dangerous character of sewer emanations. They move into a house which is tastefully papered and painted. The arrangement of the rooms is admirable, and the location, for a residence, unexceptionable. Concerning the sewerage they have never stopped to think. "It must, as a matter of course, be all right in such a fine-looking residence." People say "Our doctor has never mentioned the subject of sewer gas. If it is of such vital importance, why should he not have spoken of it?" Why, indeed? The aches and pains, and languor and lassitude which people feel are more often directly traceable to sewer-gas poisoning than is commonly believed. The question, reduced to its lowest terms, is something like this: Where there are sewers, we usually can expect to find sewer-gas. This gas is poisonous to life and health. Its disposal is a matter of the most vital importance. It must be imprisoned in a sewer or be allowed to escape into and mingle with the atmospheric air, or it may find its way into dwellings and be breathed by the inmates. Here are three ways of managing this toxic agent. The last mentioned is disastrous to health, and should be prevented. In some cities, and Chicago has made the experiment to a limited extent, the plan of ventilating sewers is in vogue. Openings in the center of the street, protected by perforated iron covers, allow the escape of sewer-gas into the atmosphere of the street, and also, at the same time, permit the ingress of atmospheric air into the sewer. To my mind, this is a most sensible plan for diluting and rendering impotent a certain amount of sewer-gas poison. It is not claimed that this method of diluting sewer-gas is sufficient to meet all the demands. The plan is good as far as it goes, but it does not do away with the necessity for traps at sinks and closets. There are at present several kinds of apparatus which prevent the escape of most of the sewer-gas contained in the waste-pipes. That they do entirely prevent the entrance of sewer-gas into living rooms is probably not true, although the inventors of the various kinds of sewer-traps claim that no gas escapes.

Sewer-gas can not follow its victim into the open air, or if it does, its power is destroyed by the admixture of atmospheric air. It is a domiciliary foe. As such it must be met, and fought, and conquered. Either shut it out, exclude it entirely, or antidote its poison by atmospheric air.

Sewer-gas is not always a rapid poison. It does not always kill. Its approach and attack are frequently so insidious and stealthy that the victim is not aware of his danger. People are often inhaling enough sewer-gas to affect the health, and at the same time unaware of its presence. This subtle and intangible character of sewer-gas is what makes it such a dangerous enemy. "Ordinary sewer-gas is composed of either or all of the following gases: Sulphureted hydrogen, carbureted hydrogen, carbonic acid and ammoniacal vapors, besides some others." Odor is not always a characteristic of sewer-gas. Physicians who are able to recognize the effects of sewer-gas poisoning, can do an incalculable benefit to their patrons by giving them timely warning. There is no need to exaggerate. The bold statement of facts is, or ought to be, enough. Diphtheria, small-pox, and scarlatina are carried on the "invisible wings" of sewer gas to different parts of the city. There can be no doubt that these formidable and ever-to-be-dreaded diseases are scattered broadcast through the agency of sewer emanations.

The blocks and flats put up in cities are frequently built *to rent.* The owner consults an architect, who draws the plans. Then a contractor undertakes the work of construction. Too often the contract is let to the "lowest bidder," without any thought of his real ability to do good work. He sub-lets the stone and brick work, and before the block is ready for occupancy a dozen or score of different firms have had a hand in the work of erecting the building. The plumber is under contract to furnish the lead pipes and to equip the building with all the necessary apparatus for carrying off the waste of domestic life, and for preventing the ingress of sewer emanations to the living rooms. Too often he employs cheap and unskillful workmen, who slight their work, and thus endanger the health of the future occupants of the building. In neglecting to make perfect joints, and to properly trap every sink and closet he is committing a crime against society of no small magnitude. The builder of a ship might better put a rotten plank into the hull, than that a plumber should do dishonest and unskillful work. The house is to be occupied for years to come.

A failure to construct properly that part of the building which is designed to carry away the refuse, is many times followed by fatal consequences. The plumber's work is, when the building is completed, nearly all concealed from view. Only a system of house inspecting by men qualified for the work, and honest and trustworthy, and above receiving "hush money" for slighting their duties and closing their eyes to imperfect plumbing, only this can save us from the dangers of breathing foul gases from sewers. If the painter or glazier, or grainer fails to take the pains, in doing his work, he ought to take, nobody's health suffers thereby. The plasterer and roofer can do shoddy work, and still not endanger human life. That there are intelligent, capable and honest plumbers no one can deny; but the rank and file of this body of useful artificers are not so skillful and efficient as they ought to be. Professor C. A. Lindsley, of Yale College, affirms that "sewer-gas is so subtle that its presence is many times not detected, and yet so laden with the germs of disease that diphtheria, scarlet fever, typhoid fever, and other fatal maladies are the sure event to those who dwell in such air-poisoned houses." He further says: "It is a species of crime to permit property owners, through ignorance, or for any other reason, to go on unrestrainedly putting numbers of our fellow-citizens in danger by connecting houses with sewers without adopting the safeguards necessary for their protection." Sewer-gas is no myth. It really exists, and is in active operation day and night, lowering the vitality and vitiating the blood of thousands of unsuspecting people. "Men who will spend thousands of dollars in painting, gilding and carving their houses, will not spend one cent to make sure that the air they and their families breathe is not reeking with dangerous impurities, which would make their hair stand on end with horror could they see them magnified a few hundred times." Doctors know something about these dangers, although not half what they ought to know. Many contend that their mission and business is to cure, not to prevent. Sewer-gas furnishes them with employment from year's end to year's end. When sickness appears, the doctor is called in at an expense of hundreds of dollars; whereas, a few dollars spent in making sure of one's drains every year would obviate the necessity of a physician. Says Professor Chandler, of New York City, the eminent chemist: "It should be thought of far more importance to clean one's drains and traps every year than to shake one's carpet and to paint one's house."

I will now give you a brief sketch of a case upon the authority of Professor Walter S. Haines:* A young man and two younger sisters were brought to their beds by sickness, and for the space of two months two physicians gave them constant attention, and all this while were absolutely ignorant of the cause of their prostration. The two girls lost for the time the senses of speech, hearing and sight, and both were attacked with with hemiplegia. The cases growing more desperate, another physician was called in. He, recognizing sewer-gas poisoning as the malady, caused the patients to be removed to another house, soon after which they began to recover, and ultimately regained their health. An examination of the drain of the house where these patients were taken sick showed that it was broken in numerous places, and gas was escaping. The main drain (usually placed under the house), frequently breaks from unequal settling of the ground on which it rests, by the weight of the walls, or because the joints are imperfect when made.

I learned the following facts in a conversation with Dr. F. A. Stanley, of this city: He was called to see a child sick with diphtheria. Two days later two more children belonging in the same family, were stricken with the same disease. He gave the cases his most assiduous attention. Nothing in the ordinary line of therapeutics was left untried. Drugs were of no avail. The children grew rapidly worse. Sewer-gas was thought of as the possible cause of all the trouble, but on account of the apparently excellent condition of the drainage, this idea was given very little attention. The children continued to grow worse. One died. The faith of the family in their physician was receiving a severe test. The second child soon followed the first. The third child was growing worse. Then, at last (and all these things just related transpired in a very few days), the fourth child was stricken down with diphtheria. Sewer-gas was once more thought of as possibly being an etiological factor in the causation of all this suffering. At an outlay of considerable

* I quote from "Sewer Gas and Its Dangers," by Geo. Preston Brown.

labor, the house drain was examined, with the expectation of finding it in good condition. On the contrary, it was found to be in a most imperfect condition. Where the waste pipe emptied into the main drain, a broken joint was discovered. The soil was saturated for yards around this leaky joint with the refuse from the kitchen and water-closet, and thus the enigma was solved. The drain was repaired and the sick children were removed to another part of the house. One recovered. Can there be any reasonable doubt that the blood-poisoning in these cases came from sewer emanations? In February of this year I was summoned to attend Mr. J. T., a salesman for a leading dry-goods firm of this city. He was strictly temperate in his habits. I found him on the third day after he had stopped work, with a temperature of 104.2-5° Fahrenheit, a rapid and somewhat feeble pulse, a tongue heavily loaded with brown fur. He said he suffered from no pain whatever. He complained principally of an almost uncontrollable feeling of languor and fatigue. Countenance pale, eyes sunken, breath offensive, perspiration excessive. I administered remedies to reduce the temperature, but succeeded only indifferently. This patient continued to grow rapidly worse, and within one week from the time of his first attack he was dead. Subsequent inquiry revealed the existence of a defective drain under the store where this unfortunate man was employed.

Additional corroborating evidence, in favor of the theory of sewer-gas poisoning, was found in the fact that several of the employés in this same store were, about this time, ill from what, as near as I could learn, was blood-poisoning. Chloride of lime had been used plentifully in the basement of this store, in the vain hope that the gods might be propitiated in that way. A liberal use of "jockey club" or "white rose" upon the person of an unwashed and malodorous tramp would be parallel treatment to this. It is probable that this man's life could have been prolonged had the necessary precautions been taken to see that the building, in which he spent more than twelve hours a day, was in proper sanitary condition.

The knowledge of this and like cases is, or ought to be, of practical benefit to every physician. The drainage and sewerage of every building, containing a patient under the care of a medical practitioner, ought to be taken into account as a possible etiological factor in the production of many diseases. Scarlatina and diphtheria are diseases which every physician dreads to encounter. If sewer-gas poisoning has to do with either the causation or fatality of these maladies, then the doctor who ignores or fails to inform himself as widely as possible upon this subject, fails to do his whole duty to his patrons. Dr. Z. P. Hanson, of this city, gives me the following notes of a case recently attended by him: He was called to attend the son of a gentleman living on the West Side. The case was diphtheria, severe from the first. Extensive ulceration and sloughing of the tissues of the throat were among the unfavorable symptoms. A servant girl, living in this same house, was about this time attacked with diphtheria in its most malignant form. Sewer-gas was strongly suspected, and though the owner of the house was inclined to be skeptical in regard to the agency of sewer-gas as a cause of the sickness of his son and servant-girl, he carried out the doctor's wishes, and employed a plumber to investigate the matter. To the surprise of the father a leaky joint was found. It had been attempted to make a curved joint out of two straight pieces of tile, by adopting the miserable, slipshod and criminal device of putting in a section of a collar, and endeavoring to join the two straight pieces of tile. The cement which had been used to close the seams in this joint had disintegrated and crumbled away, and nothing was left to hinder the contents of the drain from passing into the soil and saturating it with the house-slops. Since repairing the drain no sickness has occurred in the house, whereas, before this, some member of the household was ill a good deal of the time. This revelation of the defective character of the drain seemed to explain the probable etiology of a peculiar and fatal case of peritonitis, which had occurred in this same house some time previous to this last attack of diphtheria just mentioned.

It devolves upon every physician, practicing medicine in localities where the health of his patrons is menaced by this unseen foe, to warn the unsuspecting and put the public (or as much of the public as comes within his jurisdiction) upon its guard.

I am not insisting upon anything against which there is any considerable amount of opposition. The American Public Health Association is no longer a struggling and unpopular society, existing only through the efforts of a few enthusiastic workers. It stands upon as firm a basis as does its sister society and coadjutor, the American Medical Association.

The chemistry of sewer-gas ought to receive a passing notice. Dr. Letheby, the author of that standard work on "Food" declares that in all sewer-gas, are found sulphureted hydrogen, sulphide of ammonium, and organic matter. Beside these gases, are often present nitrogen and carbonic acid. In order to understand the deadly character of sulphureted hydrogen, it may be well to say that air, "otherwise pure, containing one part in two thousand of this noxious vapor, if breathed by birds is fatal to life." In the Luxembourg Gardens, in the city of Paris, the crows at one time became so numerous and so troublesome that they were a source of infinite annoyance to frequenters of the garden. To shoot them would endanger the safety of the people who resorted to the place. The following plan was carried out with success: The gardener was furnished with a bag or jar filled with sulphuretted hydrogen gas. Attached to this was a long, slender tube, which was slyly pushed up into a tree containing a number of crows. When the tube was in the midst of the flock, the gas was permitted to escape, and numbers of crows fell dead from the effect of the gas. "Air, $\frac{1}{200}$ part of which is sulphureted hydrogen gas, will kill dogs, if breathed by them, and one part in two hundred and fifty will end the life of a horse."

It is believed that air containing $\frac{1}{100}$ part of this noisome gas is sufficient to prove fatal to human life when inhaled for any length of time. Do you wonder that those unfortunate people who live in houses into which this deadly vapor enters are sick and languid, and unfit for active work? Is it any wonder that we hear of cases of sewer-gas poisoning in the practice of medical men? Is it not rather a marvel that the harm done and the disease induced by the inhalation of this dreadful agent, is not more widespread, not accompanied with more fatal effect than is really the case. Its known effects are, when breathed in air containing it, drowsiness, headache, vomiting, incoherence of speech, etc., "A simple trace of it in the air will often produce a debilitated condition resembling typhoid fever," says one recognized as an authority on this subject.

It is probable that sulphuretted hydrogen and ammonium sulphide, when inhaled, affect the every seat and center of vitality. The red blood globules containing the compound known as hemoglobin, are robbed of the oxygen they contain, and thus their carrying power, as distributors of this indispensable element, is destroyed.

There is no doubt in my own mind, that many cases of mental and physical fatigue, supposedly the result of over-work, are caused by inhalation of sewer-gas and air made impure from stagnation and respiration. Bright's disease of the kidneys is said to be caused by sewer-gas inhalation, on account of the high arterial pressure it produces.

Says Dr. De Verona, of Brooklyn: "Very small amounts of sewer-gas may develop fatal ailments on the one hand, and on the other hand, large amounts may produce but slight derangements, and above all, the poison once implanted in a human being is capable of reproducing itself ad infinitum."

The question arises, how shall we disinfect sewer-gas and render it inert? The use of sulphate of iron and chloride of lime and carbolic acid are practically useless in combating with poison in the form of sewer-gas. For the disinfection and deodorization of open drains and even sinks, these drugs are pre-eminently efficacious if used with thoroughness and in sufficient quantities. But to prevent the ravages of sewer-gas by disinfecting water-closets and kitchen sinks with any or all of these drugs, would be unavailing, unless, indeed, every householder in the entire district where sewers were placed should disinfect his premises. Could this be done, disinfection might be practicable. But its non-feasibility is apparent when we remember the numbers of ignorant and filthy people who live in every large city, and who would not comply with any such rule as this unless they were actually obliged to do so. We need not inhale sewer-gas in any appreciable quantity if the plumbers and sewer builders will do honest work. In England the subject of house drainage has received closer attention than in this country. Trans-Atlantic sanitarians found out years ago the dangerous character of sewer-gas, and with the thoroughness which distinguishes the English mind, have been studying out a way to banish it from their houses.

It should be borne in mind that the public, the lay members of the medical profession, derive most of their knowledge of hygiene and sanitary medicine from the physicians who are called into their families. These patrons will never think of asking in regard to the physiological action of eucalyptus globulus, or about the manner in which nux vomica affects the nervous system and digestive apparatus. But questions concerning ventilation, house drainage, diet, clothing, exercise, etc., are sure to be propounded. It should not be considered beneath the dignity of any medical practitioner to make himself familiar with all the details of house drainage and plumbing. Until this is done, he can not appreciate and measure the evils that arise from defective sewers and improperly constructed drains. It is too often the case, that men in this city, as well as in others, are licensed to build sewers and to equip dwellings with drains and waste-pipes, and traps, whose knowledge of the laws of natural philosophy is limited to the single fact that water naturally runs down hill. Until this slip-shod system of public service is done away with, and men are selected for this kind of work whose qualifications are of a higher order, we shall not see any marked improvement in house drainage.

257 *West Madison St.*

